# Medication used in intentional drug overdose in Flanders 2008-2013

**DOI:** 10.1371/journal.pone.0216317

**Published:** 2019-05-02

**Authors:** Nikita Vancayseele, Ine Rotsaert, Gwendolyn Portzky, Kees van Heeringen

**Affiliations:** 1 Unit for Suicide Research, Ghent University, Ghent, Belgium; 2 Flemish Centre of Expertise in Suicide Prevention, Ghent University, Ghent, Belgium; University of Toronto, CANADA

## Abstract

**Background:**

Intentional drug overdose is the most common method of self-harm. As psychiatric disorders are very common in self-harm patients, the medication used to treat these disorders can become the means for the self-harm act. The present study aimed at investigating an association between the use of prescribed medication (analgesics and antipyretics, anti-epileptics, antipsychotics, antidepressants and psychostimulants) as a method of self-harm and prescription rates of this medication in Flanders. We investigated the possible effect of gender, alcohol use during the self-harm act and a history of self-harm.

**Methods:**

Data from the multicenter study of self-harm in Flanders between 2008 and 2013 were used. The significance of differences in percentages was calculated by GEE and the strength by odds ratios (OR).

**Results:**

There was an increase in the odds of using antidepressants (0.8%) and antipsychotics (2%) among females when the rate of prescription increases. Analgesics and antipyretics (39.3/1,000) and antidepressants (124.9/1,000) were the most commonly prescribed drugs among females. Antidepressants (63.9/1,000) and antipsychotics (26.5/1,000) were the most commonly prescribed drugs among males. Antidepressants and analgesics and antipyretics were the most frequently used medications for self-harm. Analgesics and antipyretics during the self-harm act were more common among first-timers, while repeaters more commonly overdosed using antipsychotics and antidepressants.

**Conclusion:**

These findings suggest that the availability of medication via prescriptions plays an important role in the choice of the medication ingested during the self-harm act. Precautions are necessary when prescribing medication, including restrictions on the number of prescriptions and the return of unused medication to pharmacies after cessation of treatment. These issues should be a focus of attention in the education and training of physicians and pharmacists.

## Introduction

Suicidal behaviour continues to pose major challenges to (mental) health care. Suicidal behaviour is closely associated with mental health problems, with the most common being depression, substance use and anxiety disorders [1–4], so their treatment can be expected to contribute substantially to suicide prevention. However, the effect of psychotropic medication on suicidal behaviour is a matter of debate since decades. The possibility that the use of antidepressants may lead to an increase in suicidal behaviour has led the Food and Drug Administration (FDA) to introduce a black-box warning, following which their use decreased but suicide rates increased in particular age groups [5]. In addition, while psychotropic drugs have an established efficacy in treating psychiatric disorders, early pharmaco-epidemiological studies have suggested a positive correlation between rates of their prescription and those of their use in non-fatal suicidal behaviour [6, 7]. A recent study showed that subjects using a psychotropic drug during a self-harm act are more likely to have had recent prescribed access to any psychotropic drug and to the drug type used prior to the self-harm episode when compared to those not using any psychotropic drug in a self-harm episode [8]. A better understanding of the link between rates of drug prescriptions and those of self-harm acts can be expected to contribute substantially to the prevention of suicide. For every suicide there are many more people who attempt suicide every year [9]. A prior self-harm act is the single most important risk factor for suicide in the general population and intentional drug overdose is the most common method of self-harm in people who present to hospital, being used in 68 to 80% of self-harm episodes [10–16]. Previous studies have documented a positive association between alcohol use and self-harm [17, 18]. Consuming alcohol within six hours of and as part of the act of self-harm increases the lethality of acts of self-poisoning, e.g. those involving paracetamol, co-proxamol and sedatives [19]. The majority of self-harm cases involve their own home medication or prescribed medications, while over-the-counter (OTC) medications are less likely to be chosen for self-harm acts [20–23]. Given these consistent epidemiological findings it can be hypothesized that the rate of prescribing of medication influences the occurrence and characteristics of non-fatal suicidal behaviour. In addition, the association between the use of prescribed medication during a self-harm act and prescription rates in Flanders has not yet been investigated. This study extends previous research by utilizing two large Flemish monitoring systems.

The present study therefore aimed at investigating an association between the use of prescribed medication as a method of self-harm and prescription rates of these medications in Flanders. We hypothesized that an increase in rates of prescribing is associated with an increase in the number of non-fatal self-harm acts with that medication. We thereby investigated the possible effects of gender and also the effects of alcohol use during the self-harm act and a history of self-harm as they are strongly associated with suicidal behaviour [17, 24, 25].

## Method

### Definitions

The general term suicidal behaviour refers to a range of thoughts and behaviours i.e. suicide ideation, deliberate self-harm, attempted suicide and suicide [26]. The term ‘attempted suicide’ is commonly used for episodes in which there was at least some suicidal intent (i.e. a wish to die). The term ‘deliberate self-harm’ is often used regardless of intent which implies that ascribing intent is avoided rather than implying a lack of intent [27]. In this paper we used the term ‘self-harm’, as this term refers to self-injury or intentional self-poisoning regardless of apparent motivation or the extent of suicidal intent [28, 29]. We prefer to use the term ‘self-harm’ instead of ‘deliberate self-harm’ because the prefix ‘deliberate’ is judgmental while the extent to which the behaviour is intentional is not always clear [28]. The monitoring study uses the following definition of self-harm: “an act with nonfatal outcome, in which an individual initiates a deliberate, well-considered, and unusual behaviour, that without intervention of another will lead to self-harm or destruction, or when an individual deliberately takes a substance in higher quantity then subscribed or generally suitable doses, with the intention by means of actual expected physical consequences to initiate desired changes” [30]. This definition was developed by the WHO/Euro Multicentre Study.

### Catchment area and population under study

Flanders is a district in the northern part of Belgium, bordering with The Netherlands, Germany and France. In 2013 there were 6,381,859 inhabitants in Flanders, including 3,151,466 males. 3,938,104 inhabitants were aged between 18 and 64 years. In 2013, 51% of the Flemish people indicated the experience of psychological problems, the most common are sleep problems, anxiety problems, burnout and mood problems [31]. The Flemish suicide rates were 1.5 times higher than the European mean suicide rate in 2013. In Flanders, in the year 2013, the total suicide rate per 100,000 inhabitants was 16.3 (in comparison, rates were 6.3 and 32.6 in Italy and in Lithuania, respectively) [32]. Compared to self-harm rates found in other monitoring studies such as Ireland (199/100,000) [33] and Oxford (293/100,000) [34], Flanders had lower self-harm rate in 2013 (156/100,000) [35].

### Ethics statement

The ethical committee of the University hospital in Ghent approved the study (titled: De epidemiologie van suïcidepogingen in Vlaanderen: 2008/675) according to the principals expressed in the Declaration of Helsinki. Data were analyzed anonymously.

### Prescriptions

The IMA (agency of the Belgian health insurance; Intermutualistisch Agentschap) supplied the data on the prescriptions of drugs in Flanders belonging to five ATC (Anatomical Therapeutic Chemical Classification) codes including (1) N02B analgesics and antipyretics (including paracetamol and acetylsalicylic acid) (2) N03A anti-epileptics (including phenytoin, carbamazepine, valproate and pregabalin); (3) N05A antipsychotics (which included haloperidol and pimozide (4) N06A antidepressants (which included SSRI antidepressants such as citalopram, sertraline, escitalopram and tricyclic antidepressants such as clomipramine, imipramine, doxepin) and (5) N06B psychostimulants, including agents used for ADHD and nootropics (which include atomoxetine and methylphenidate). The IMA provided information about the number of people who received a prescription of the specified medications. Rates of prescribing were calculated per 1,000 population using annual population estimates from 2008–2013. For example, in 2013, 620,337 persons of the 6,365,598 Flemish inhabitants (97.45 persons per 1,000 inhabitants) received a prescription for an antidepressant.

### Self-harm

We used data from the monitoring study of self-harm in Flanders on individuals who presented with self-harm to the emergency departments of 31 General Hospitals and two University Hospitals between 1^St^ January 2008 and 31^st^ December 2013. Data collection began in 2008 in 5 hospitals. The number of participating hospitals gradually increased over the years, and in 2013 there were 33 participating hospitals. In 2013, there was a 47.7% coverage of the hospitals in the five counties of Flanders including two University Hospitals and 31 General Hospitals. The University Hospitals have more than 80,000 hospital admissions per year. The number of hospital admissions in the General Hospitals varies between 10,000 and 35,000 per year. All participating hospitals have an emergency department and 23 hospitals have a psychiatric department. A semi-structured interview is used by clinicians and nurses as part of a psychosocial assessment. In this study we used data collected by means of this interview. The dataset includes information on demographics (age, gender), characteristics of the self-harm act (including which drug(s) were ingested in overdose), and history of self-harm. Patients who overdosed on medication from the described ATC groups at any time during the study period, either alone or in combination with other substances, or in combination with self-injury, were selected from this population. Event-based data rather than person-based data were used in the analyses, as the study focused on the ingestion of specific drugs rather than on personal or clinical characteristics.

### Statistical analyses

Prescription rates (numbers of prescriptions per 1,000 population) were calculated for each year (2008–2013) for the five drug groups using prescription and population data for Flanders. Frequencies were calculated to describe the sample. All statistical analyses were performed in R version 3.4.1. Using Generalized Estimating Equations (GEE), the correlation in response was accounted for by modelling the covariance pattern. By specifying an exchangeable covariance pattern, we assumed that all correlations are the same. Robust standard errors for the estimates were used to compute the 95% confidence intervals. For the GEE analysis the subjects are nested within years and provinces, the variables *history of self-harm*, *alcohol use* (both categorical variables) and *prescription rates* (continue variable) were used as covariates and *gender* as between subjects factor. The 95% confidence intervals (CI) of the Odds Ratio (OR) are described. Data analysis was carried out relating to all self-harm acts in which intentional drug overdose was involved with medication with the following ATC codes N02B (analgesics and antipyretics), N03A (anti-epileptics), N05A (antipsychotics), N06A (antidepressants) and N06B (psychostimulants).

## Results

During the study period, the monitoring system reported 9,487 episodes of self-harm in the participating hospitals in Flanders between 2008 and 2013 (Table 1). The majority of self-harm patients were female (61%). The mean age for both genders was 39 years. Among the 9,487 self-harm acts, 7,388 (78%) involved self-poisoning only (overdose medication and/or self-poisoning with pesticides or gases and unspecified chemicals and noxious substances), while 1,622 involved self-injury only and 471 involved both self-poisoning and self-injury. For 6 episodes, the used method was unknown. The ingestion of an overdose of medication accounted for 81% of the self-harm episodes. Females reported significant more intentional drug overdose during the self-harm act than males (86% vs 72%; χ^2^ = 320.42, p < .01). In 6.2% of the self-harm episodes, multiple medications with different ATC codes were used, significantly more common among females than males (6.9% vs 5.2%; χ^2^ = 11.52, p < .01). Antidepressants in combination with antipsychotics were used in 2.7% (n = 259) of the self-harm acts. The second most used combination were analgesics and antipyretics and antidepressants (1.7%; n = 40). In 0.5% (n = 21) of the self-harm episodes three types of medication were used during the act for example anti-epileptics in combination with antipsychotics and antidepressants or analgesics and antipyretics in combination with antidepressants and psychostimulants. In 29% of the self-harm episodes alcohol was involved during the self-harm act, significantly more common among males than among females (34% vs 26%; χ^2^ = 68.75, p < .01). Significantly more females than males had a history of self-harm episodes (56% vs 47% χ^2^ = 53.83, p < .01).

**Table 1:** Descriptive results of self-harm acts in Flanders from 2008 to 2013 by gender.

	Male	Female	All
**Gender ^(a)^**	39% (3,672/9,478)	61% (5,806/9,478)	100% (9,478/9,478)
**Mean Age (Sd)**	38.6 (14.9)	39.2 (15.8)	39 (15.5)
	**% [95% CI] (n/3,699)**	**% [95% CI] (n/5,803)**	**% [95% CI] (n/9,481)**
**Self-poisoning ^(b)^**	69% [67.4, 70.4] (2,527)	84% [82.4, 84.6] (4,854)	78% [77.1, 78.8] (7,388)
**Self-injury ^(b)^**	25% [23.9, 26.7] (927)	12% [11.1, 12.8] (693)	17% [16.4, 17.9] (1,622)
**Both ^(b)^**	6% [5.2, 6.7] (215)	4% [3.9, 5] (256)	5% [4.6, 5.4] (471)
**Overdose medication ^(b)^**	72% [70.1, 73] (2,626)	86% [85.6, 87.3] (5,018)	81% [79.8, 81.4] (7,644)
**Use of alcohol**	34% [32.8, 35.9] (1,259)	26% [25.2, 27,4] (1,524)	29% [28.5, 30,3] (2,783)
**Previous self-harm episode(s) ^(c)^**	47% [45.4, 49] (1,410)	56% [54.3, 57.2] (2,647)	52% [51.3, 53.6] (4,057)
**% patients on multiple medications with different ATC codes ^(b)^**	5.2% [4.5, 5.9] (190)	6.9% [6.2, 7.5] (401)	6.2% [5.7, 6.7] (591)

^(a)^ For 9 episodes gender was not known, thus total number of episodes 9,487

^(b)^ Method unknown for 3 males and 3 females

^(c)^ Previous self-harm episodes unknown for 682 males and 1,056 females

**Fig 1:**
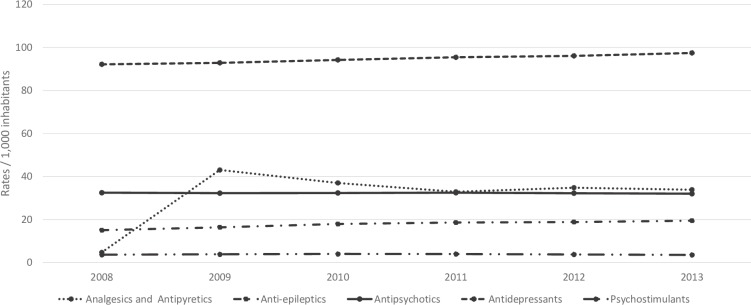
Trends in prescription rates for the five ATC groups.

Fig 1 shows the trends in prescription rates (e.g. the number of people who received a prescription of the specified medication) for the five ATC groups. The prescription data for Flanders showed a significant increase in prescription rates of analgesics and antipyretics (χ^2^ = 30.82, p < .001) from 2008 to 2009 from 4.75 (95% CI 2–11.3) to 43.06 (95% CI 32.1–57.5) per 1,000 inhabitants. The prescription rate for analgesics and antipyretics decreased (not significantly) with 21% from 43.06 (95% CI 32.1–57.5) in 2009 to 33.88 (95% CI 24.3–47) in 2013. The prescription rates per 1,000 inhabitants of antidepressants are the highest between 2008 and 2013 compare to the other drug groups and increased (not significantly) with 5% from 92.17 (95% CI 75.8–111.7) in 2008 to 97.45 (95% CI 80.6–117.4) in 2013. Psychostimulants have been prescribed the least often compared to analgesics and antipyretics, anti-epileptics, antipsychotics and antidepressants. The prescription rate for psychostimulants and antipsychotics remain stable between 2008 and 2013.

The prescription rates for analgesics and antipyretics, anti-epileptics, antipsychotics, antidepressants and psychostimulants in the general population and the percentage of self-harm acts where intentional drug overdose was used as method (e.g. 81% of the self-harm acts) are shown in Table 2. The median of prescription rates was higher among females than among males for four out of five ATC medication groups. The median for medication with code N06A (antidepressants) was almost two times higher among females (124.9/1,000) than among males (63.9/1,000). Antidepressants were the most commonly prescribed medication among both females and males. The median for medication with ATC code N06B (psychostimulants) was more than 3 times higher among males (5.7/1,000) than among females (1.5/1,000). From 2008 to 2013, the largest proportion of intentional drug overdoses could be attributed to medication with ATC code N06A (antidepressants) and N02B (other analgesics and antipyretics) during self-harm acts with intentional drug overdose in both males (20% and 16%, respectively) and females (25% and 17%, respectively). During the study period, females (25%) significantly ingested antidepressants more commonly (N06A) during a self-harm act involving intentional drug overdose than males (20%; χ^2^ (1) = 12.68, p < .001). The median of prescription rates for anti-epileptics (N03A) was higher among females (18.9) than among males (15.9), yet males ingested this medication marginally significantly more commonly during a self-harm act with intentional drug overdose than females (3% vs 2%; χ^2^(1) = 3.63 p = .057). Overall, the anti-epileptics (N03A) were less commonly ingested in overdose than their use in the community would predict, in both males (3%) and females (2%).

**Table 2:** Rate of prescriptions, percentage of self-harm episodes with intentional drug overdose as method in Flanders from 2008 to 2013.

ATC	Rate/1,000 median (25^th^ perc– 75^th^ perc)	% self-harm episodes with intentional drug overdose as method [95% CI] (n/N)
**MALE**		
**Analgesics and antipyretics**	24.5 (25.9–28.8)	16% [14–17] (413/2626)
**Anti-epileptics**	15.9 (17.6–18.9)	3% [2–4] (75/2626)
**Antipsychotics**	26.5 (26.9–31.2)	13% [12–15] (349/2626)
**Antidepressants**	63.9 (65.7–72.3)	20% [19–22] (526/2626)
**Psychostimulants**	5.7 (6–7.2)	1% [1–2] (28/2626)
**FEMALE**		
**Analgesics and antipyretics**	39.3 (41.8–44.3)	17% [16–18] (845/5018)
**Anti-epileptics**	18.9 (20.3–22.3)	2% [2–3] (107/5018)
**Antipsychotics**	35.5 (36.4–42.1)	12% [11–13] (616/5018)
**Antidepressants**	124.9 (127.8–136.2)	25% [23–26] (1230/5018)
**Psychostimulants**	1.5 (1.6–1.9)	1% [1–1] (36/5018)

Table 3 shows the odds ratios of using particular medication in self-harm episodes among males and females. A particularly relevant finding is that among females who self-harm, the odds of using antidepressants during the self-harm act were expected to increase with 0.8% when the rate of prescription increases by 1 prescription/1,000 inhabitants (95%CI 1–1.015). Among males who self-harm, the odds of using antipsychotic medication during the self-harm act was expected to increase with 5% when the rate of prescription increases by 1 prescription /1,000 inhabitants (95% CI 1.017–1.088). Among females who self-harm, the odds of using antipsychotic medication during the self-harm act was expected to increase with 2% when the rate of prescription increases by 1 prescription/ 1,000 inhabitants (95% CI 1.006–1.034).

**Table 3:** Odds ratio due to specific drug categories, prescription rates, alcohol use and history of self-harm per gender.

		Male			Female		
		OR	(LCL-UCL)	p	OR	(LCL-UCL)	p
**analgesics and antipyretics**	Rate	0.988	(0.971–1.004)	0.146	0.996	(0.991–1)	0.073
	Alcohol use	1.363	(1.104–1.683)	0.004	0.803	(0.625–1.032)	0.087
	History of self-harm	0.719	(0.569–0.922)	0.009	0.737	(0.612–0.887)	0.001
**anti-epileptics**	Rate	1.019	(0.966–1.076)	0.485	1.112	(0.99–1.248)	0.073
	Alcohol use	0.712	(0.361–1.406)	0.328	0.54	(0.32–0.91)	0.021
	History of self-harm	0.922	(0.494–1.719)	0.798	1.99	(1.284–3.084)	0.002
**antipsychotic**	Rate	1.052	(1.017–1.088)	0.003	1.02	(1.006–1.034)	0.006
	Alcohol use	0.739	(0.55–0.933)	0.045	0.823	(0.673–1.007)	0.059
	History of self-harm	2	(1.511–2.646)	0	2.409	(1.976–2.937)	0
**antidepressants**	Rate	1.005	(0.991–1.02)	0.498	1.008	(1–1.015)	0.047
	Alcohol use	1.31	(1.119–1.533)	0.001	1.093	(0.901–1.327)	0.365
	History of self-harm	1.581	(1.244–2.008)	0	1.476	(1.315–1.657)	0
**Psychostimulants**	Rate	0.708	(0.46–1.091)	0.118	0.32	(0.098–1.045)	0.059
	Alcohol use	1.616	(0.734–3.513)	0.226	0.741	(0.338–1.623)	0.454
	History of self-harm	1.103	(0.528–2.305)	0.794	1.253	(0.645–2.435)	0.505

Table 3 also shows the results of the Generalized Estimating Equation for the five medication groups, per gender, with alcohol use during the self-harm act, and with a history of self-harm. When males used alcohol during the self-harm act, the odds of using analgesics and antipyretics during the self-harm act increased significantly by 36% (OR = 1.363, p < .01). Among females there was no such significant effect (OR = 0.803, >.05). The odds of using analgesics and antipyretics during a self-harm act increased significantly when there was no history of self-harm episodes, 28% in males (OR = 0.719, p < .01) and 26% in females (OR = 0.737, p < .01).

The odds of using anti-epileptics during a self-harm act increased significantly when females used no alcohol during the self-harm act (OR = 0.54, p < .05) and when there was a history of self-harm episodes (OR = 1.99, p < .01). Among males there was no such significant effect.

The odds of using antipsychotic during a self-harm act increased significantly when males used no alcohol during the self-harm act (OR = 0.739, p < .05) and when there was a history of self-harm episodes (OR = 2, p < .001). The odds of using antipsychotic during a self-harm act increased marginally significant when females used no alcohol during the self-harm act (OR = 0.823, p = .059) and when there was a history of self-harm episodes (OR = 2.409, p < .001).

The odds of using antidepressants during a self-harm act increased significantly when males used alcohol during the self-harm act (OR = 1.31, p < .01) and when there was a history of self-harm episodes (OR = 1.581, p < .001). The odds of using antidepressants during a self-harm act increased significantly when females had a history of self-harm episodes (OR = 1.476, p < .001).

There were no significant results with regard to the odds for using psychostimulants, neither in males nor in females.

## Discussion

As far as known to the authors this is the first study of the association between rates of prescription of particular drugs and those of their use as a method of self-harm during an intentional drug overdose at a population level. The results can be summarized as follows. Between 2008 and 2013, antidepressants and analgesics and antipyretics are the most commonly prescribed drugs in females, while antidepressants and antipsychotics were the most prescribed drugs in males. Antidepressants and analgesics and antipyretics are the most commonly ingested drugs during a self-harm episode using intentional drug overdose. Analgesics and antipyretics are more commonly used in first self-harm episodes, while overdoses of antidepressants are more commonly involved in repeaters. Antidepressants are significantly more commonly used in intentional drug overdose among females than among males. These results are very similar to those of the study of Townsend et al [36] and Tournier et al [21]. Non-fatal suicidal behaviour may be facilitated by the used medications through means other than the prescribed medications, a study has shown that a large proportion of those who had used violent methods had combined suicide method with poisoning. For example, more than half of those who had completed suicide by drowning had also taken other substances such as alcohol and opioids [37].

Preceding a discussion of these findings in terms of their relevance for the prevention of suicidal behaviour a few methodological issues need to be addressed. The study was carried out in subjects admitted to general hospitals, therefore the self-harm study group included no subjects with self-harm who were treated in other facilities or medically unidentified. We also have no information about patients who needed an intensive or surgical procedure following their severe self-harm act, as they were not seen by a psychologist/psychiatrist directly after admission. This indicates a possible under reporting of the real number of people who self-harm in Flanders. Since the number of self-harm acts involved is large, the findings are likely to be representative. This limitation may have a bias on the types of medication that was used during the self-harm act. The patients who were not admitted at the emergency department after their self-harm episode may have taken a different kind of medication compared to the patients who were admitted. The over-the-counter availability of analgesics and antipyretics was not taken into account in this study. The presence of analgesics and antipyretics is probably underestimated given that these drugs are widely available without prescriptions. The types of medication ingested during the self-harm act were identified during the psychosocial assessment with the patient and not using objective measurements, such as a blood test. The accuracy of the results thus depends on the patient statements. As a final limitation of this study, we can state that the semi-structured interview that was used during the psychosocial assessment is not a standardized and validated instrument.

### Implications for suicide prevention

Numerous factors contribute to the occurrence of suicide which is never the consequence of one single cause of stressor. The Integrated Motivational- Volitional (IMV) model of suicidal behaviour is a tri-partite model that describes the biopsychosocial context in which suicidal ideation and behaviour may emerge, the factors that lead to the emergence of suicidal ideation and the factors that govern the transition from suicidal ideation to suicide attempts/ death by suicide [38]. The final phase of the IMV model outlines the factors (volitional moderators; VMs) that govern the transition from suicidal ideation to enaction. VMs can be environmental and having access to means of suicide is an important risk factor for suicide [38, 39]. Access to means for self-harm or suicide is an important component of suicide prevention, and a limitation in such access has been clearly shown to reduce rates of suicidal behaviour [40]. Since this study shows an increase in annual rates of prescription of antidepressants and in their ingestion during self-harm episodes, particularly among repeaters, the availability of medication indeed appears to be an important given for the prevention of self-harm. Physicians have to take in consideration that prescribing medication contributes to this availability. Depression is a key risk factor for repetition of self-harm [41] and it is therefore likely that repeaters have been prescribed antidepressants more commonly than first-ever self-harmers. The latter may have thus less access to prescribed medication and therefore more commonly use analgesics and antipyretics, which are easily available without prescription. The association between antidepressant prescriptions and their use in self-harm does not implicate that taking such medication lead to an increase in self-harming behaviour. Given the link between self-harm and psychiatric disorders, and depression in particular, the availability of antidepressants will be greater in individuals who self-harm than in the general population (probably in a similar way as patients who die from a heart attack more commonly will have taken cardiology medication).

Antipsychotic drugs are the third most commonly used medication during an episode of intentional drug overdose, and an increase in their use was paralleled by an increase in the number of prescriptions of antipsychotic medication in Flanders. Such a positive correlation is not found for analgesics and antipyretics, which are the second most commonly used drugs for self-harm. This is probably due to the fact that these drugs are widely available without the need for a prescription.

The current results underline the importance of the availability of medication for the prevention of suicidal behaviour. The beneficial effects of limiting the availability of medication on the occurrence of suicidal behaviour have already been shown elsewhere, including restrictions in the number of prescriptions and prescribed tablets and complete withdrawal as a prevention strategy in Ireland, the UK and Denmark [42–46]. It should be noted that these examples involve analgesics medications that are toxic in overdose, and the examples thus do not generalize to psychotropic medications. In effect, it would be potentially dangerous to recommend reducing or withholding such psychotropic medications on the basis of a plan to reduce suicide rates as this could well lead to an increase in suicide rates [47–49]. In addition, more recently developed psychotropics such as SSRIs are relatively safe when taken in overdose without alcohol or other medication [50]. Nevertheless, the current findings suggest an important role for physicians and pharmacies in this context. Prescribing small amounts of medication and close monitoring of their intake are crucial. Returning unused medication to the pharmacy is advisable [21]. Physicians should be aware of other medications their patients have access to [51]. Prescription Drug Monitoring Programs (PDMP) as developed in the US provide a good example of a database which contains information about the dose, supply and prescriber of scheduled drugs the patient has filled and which is accessible to prescribing physicians and to pharmacies [52, 53]. Training, particularly of physicians who deal with patients at high risk of suicide, can also be an effective method to influence prescribing behaviour and to highlight the importance of collaboration between colleagues and health services [54, 55]. Moreover, consideration should be given to psychotherapy, such as cognitive therapy, problem-solving therapy or interpersonal psychotherapy (whether or not in combination with antidepressants), its efficacy in preventing repetition of self-harm has been clearly demonstrated [40].

## Conclusion

This study shows an increase in the odds of using antidepressants and antipsychotics in episodes of self-harm when the rate of their prescription increases. While analgesics and antipyretics and antidepressants are the most commonly prescribed drugs among females and antidepressants and antipsychotics are the most prescribed medication among males, antidepressants and analgesics and antipyretics are the most frequently used drugs in episodes of self-harm. While analgesics and antipyretics are most commonly ingested during first episodes of self-harm, antipsychotics and antidepressants are more commonly used by repeaters. These findings suggest that the availability of medication ingested during the act of self-harm. Consequently, there are a number of implications for the prevention of suicidal behaviour: the number of prescriptions and tablets should be limited and the return of unused medication to pharmacies is advisable. These issues should be addressed in the education and training of physicians and pharmacies.

## References

[1] Haw C, Hawton K, Houston K, Townsend E. Psychiatric and personality disorders in deliberate self-harm patients. Br J Psychiatry. 2001;178: 48–54. 11136210 10.1192/bjp.178.1.48

[2] Chandrasekaran R, Gnanaseelan J, Sahai A, Swaminathan RP, Perme B. Psychiatric and personality disorders in survivors following their first suicide attempt. Indian journal of psychiatry. 2003;45: 45–8. 21206833 PMC2952146

[3] Beautrais AL, Joyce PR, Mulder RT, Fergusson DM, Deavoll BJ, Nightingale SK. Prevalence and comorbidity of mental disorders in persons making serious suicide attempts: a case-control study. The American journal of psychiatry. 1996;153: 1009–14. 10.1176/ajp.153.8.1009 8678168

[4] Singhal A, Ross J, Seminog O, Hawton K, Goldacre MJ. Risk of self-harm and suicide in people with specific psychiatric and physical disorders: comparisons between disorders using English national record linkage. Journal of the Royal Society of Medicine. 2014;107: 194–204. 10.1177/0141076814522033 24526464 PMC4023515

[5] Olfson M, Marcus SC, Druss BG. Effects of Food and Drug Administration warnings on antidepressant use in a national sample. Archives of general psychiatry. 2008;65: 94–101. 10.1001/archgenpsychiatry.2007.5 18180433

[6] Forster DP, Frost CEB. Medicinal Self-Poisoning and Prescription Frequency. Acta Psychiatrica Scandinavica. 1985;71: 567–74. 3927659 10.1111/j.1600-0447.1985.tb02550.x

[7] Crombie IK, McLoone P. Does the availability of prescribed drugs affect rates of self poisoning? The British journal of general practice: the journal of the Royal College of General Practitioners. 1998;48: 1505–6. 10024711 PMC1313200

[8] Brown TL, Gutierrez PM, Grunwald GK, DiGuiseppi C, Valuck RJ, Anderson HD. Access to Psychotropic Medication via Prescription Is Associated With Choice of Psychotropic Medication as Suicide Method: A Retrospective Study of 27,876 Suicide Attempts. The Journal of clinical psychiatry. 2018;79. 10.4088/JCP.17m11982 30418710

[9] World Health Organization (WHO). Factsheet Suicide. Available from: http://www.who.int/mediacentre/factsheets/fs398/en/. (accessed October 15, 2018).

[10] Doshi A, Boudreaux ED, Wang N, Pelletier AJ, Camargo CA. National study of US emergency department visits for attempted suicide and self-inflicted injury, 1997–2001. Annals of Emergency Medicine. 2005;46(4):369–75. 10.1016/j.annemergmed.2005.04.018 16183394

[11] Bergen H, Hawton K, Waters K, Cooper J, Kapur N. Epidemiology and trends in non-fatal self-harm in three centres in England: 2000–2007. British Journal of Psychiatry. 2010;197(6):493–8. 10.1192/bjp.bp.110.077651 21119156

[12] Geulayov G, Kapur N, Turnbull P, Clements C, Waters K, Ness J, et al Epidemiology and trends in non-fatal self-harm in three centres in England, 2000–2012: findings from the Multicentre Study of Self-harm in England. BMJ Open. 2016;6(4):e010538 10.1136/bmjopen-2015-010538 27130163 PMC4854013

[13] Perry IJ, Corcoran P, Fitzgerald AP, Keeley HS, Reulbach U, Arensman E. The incidence and repetition of hospital-treated deliberate self harm: findings from the world's first national registry. PLoS One. 2012;7(2):e31663 10.1371/journal.pone.0031663 22363700 PMC3282760

[14] Ting SA, Sullivan AF, Boudreaux ED, Miller I, Camargo CA. Trends in US emergency department visits for attempted suicide and self-inflicted injury, 1993–2008. General Hospital Psychiatry. 2012;34(5):557–65. 10.1016/j.genhosppsych.2012.03.020 22554432 PMC3428496

[15] Vancayseele N, Portzky G, van Heeringen K. Increase in Self-Injury as a Method of Self-Harm in Ghent, Belgium: 1987–2013. PLoS One. 2016;11(6):e0156711. 10.1371/journal.pone.0156711 PMC4889035 27249421

[16] Michel K, Ballinari P, Bille-Brahe U, Bjerke T, Crepet P, De Leo D, et al Methods used for parasuicide: results of the WHO/EURO Multicentre Study on Parasuicide. Soc Psychiatry Psychiatr Epidemiol. 2000;35(4):156–63. 10868080 10.1007/s001270050198

[17] Cooper J, Kapur N, Webb R, Lawlor M, Guthrie E, Mackway-Jones K, et al Suicide after deliberate self-harm: A 4-year cohort study. American Journal of Psychiatry. 2005;162(2):297–303. 10.1176/appi.ajp.162.2.297 15677594

[18] Crawford MJ, Csipke E, Brown A, Reid S, Nilsen K, Redhead J, et al The effect of referral for brief intervention for alcohol misuse on repetition of deliberate self-harm: an exploratory randomized controlled trial. Psychological Medicine. 2010;40(11): 1821–8. 10.1017/S0033291709991899 20047702

[19] Haw C, Hawton K, Casey D, Bale E, Shepherd A. Alcohol dependence, excessive drinking and deliberate self-harm: trends and patterns in Oxford, 1989–2002. Social psychiatry and psychiatric epidemiology. 2005;40: 964–71. 10.1007/s00127-005-0981-3 16341616

[20] Buykx P, Loxley W, Dietze P, Ritter A. Medications used in overdose and how they are acquired—an investigation of cases attending an inner Melbourne emergency department. Aust N Z J Public Health. 2010;34: 401–4. 10.1111/j.1753-6405.2010.00573.x 20649781

[21] Tournier M, Grolleau A, Cougnard A, Molimard M, Verdoux H. Factors associated with choice of psychotropic drugs used for intentional drug overdose. Eur Arch Psychiatry Clin Neurosci. 2009;259: 86–91. 10.1007/s00406-008-0839-2 18806918

[22] Lo A, Shalansky S, Leung M, Hollander Y, Raboud J. Patient characteristics associated with nonprescription drug use in intentional overdose. Canadian journal of psychiatry Revue canadienne de psychiatrie. 2003;48: 232–6. 10.1177/070674370304800406 12776389

[23] Corcoran P, Heavey B, Griffin E, Perry IJ, Arensman E. Psychotropic medication involved in intentional drug overdose: implications for treatment. Neuropsychiatry. 2013;3.

[24] Hawton K, van Heeringen K. Suicide. Lancet. 2009; 373: 1372–81. 10.1016/S0140-6736(09)60372-X 19376453

[25] Hawton K, Bergen H, Cooper J, Turnbull P, Waters K, Ness J, et al Suicide following self-harm: Findings from the Multicentre Study of self-harm in England, 2000–2012. Journal of Affective Disorders. 2015;175: 147–51. 10.1016/j.jad.2014.12.062 25617686

[26] O'Connor RC, Nock MK. The psychology of suicidal behaviour. Lancet Psychiat. 2014;1: 73–85. 10.1016/S2215-0366(14)70222-6 26360404

[27] Self-harm Skegg K. Lancet (London, England). 2005;366: 1471–83. 10.1016/S0140-6736(05)67600-3 16243093

[28] Kapur N, Cooper J, O'Connor RC, Hawton K. Non-suicidal self-injury v. attempted suicide: new diagnosis or false dichotomy? The British journal of psychiatry: the journal of mental science. 2013;202: 326–8. 23637107 10.1192/bjp.bp.112.116111

[29] Hawton K, Hall S, Simkin S, Bale L, Bond A, Codd S, et al Deliberate self-harm in adolescents: a study of characteristics and trends in Oxford, 1990–2000. J Child Psychol Psychiatry. 2003;44: 1191–8. 14626459 10.1111/1469-7610.00200

[30] Bille-Brahe U, Schmidtke A, Kerkhof AJFM, De Leo D, Lönnqvist J, Platt S. Background and introduction to the study In: Kerkhof AJFM, Schmidtke A, Bille-Brahe U, De Leo D, Lönnqvist J, editors. Attempted suicide in Europe: Findings from the multicenter study on parasuicide by the WHO Regional Office for Europe: Leiden: DSWO Press; 1994 p. 3–15.

[31] Coppens E Vermeulen B, Neyens I, Van Audenhove C. Stigmatisering t.a.v. psychologische problemen Ervaringen en attitudes in Vlaanderen. KU Leuven, 2014.

[32] Vlaams Agentschap Zorg en Gezondheid aieo. Zelfdoding: Vlaanderen in Europa (2013–2015). Available from: http://www.zorg-en-gezondheid.be/cijfers/. (accessed February 22, 2019).

[33] Griffin E, Arensman E, Corcoran P, Wall A, Williamson E, Perry IJ. National registry of deliberate self-harm Ireland, Annual report 2013. 2014 2014.

[34] Hawton K, Casey D, Bale E, Ryall J, Geulayov G, Brand F. Self-harm in Oxford 2013. 2015 2015. Report No.

[35] Vancayseele N van Landschoot R, Portzky G, van Heeringen C. De epidemiologie van suïcidepogingen in Vlaanderen; jaarverslag 2013. 2014.

[36] Townsend E, Hawton K, Harriss L, Bale E, Bond A. Substances used in deliberate self-poisoning 1985–1997: trends and associations with age, gender, repetition and suicide intent. Social psychiatry and psychiatric epidemiology. 2001;36: 228–34. 11515700 10.1007/s001270170053

[37] Stenbacka M, Samuelsson M, Nordstrom P, Jokinen J. Self-poisoning across ages in men and women—Risk for suicide and accidental overdoses. Suicidol Online-Sol. 2017;8.

[38] O'Connor RC, Kirtley OJ. The integrated motivational-volitional model of suicidal behaviour. Philos Trans R Soc Lond B Biol Sci. 2018;373. 10.1098/rstb.2017.0268 PMC6053985 30012735

[39] Hawton K, Saunders KE, O'Connor RC. Self-harm and suicide in adolescents. Lancet (London, England). 2012;379: 2373–82. 10.1016/S0140-6736(12)60322-5 22726518

[40] Zalsman G, Hawton K, Wasserman D, van Heeringen K, Arensman E, Sarchiapone M, et al Suicide prevention strategies revisited: 10-year systematic review. Lancet Psychiat. 2016;3: 646–59. 10.1016/S2215-0366(16)30030-X 27289303

[41] Hawton K, Kingsbury S, Steinhardt K, James A, Fagg J. Repetition of deliberate self-harm by adolescents: the role of psychological factors. Journal of adolescence. 1999;22: 369–78. 10.1006/jado.1999.0228 10462427

[42] Simkin S, Hawton K, Sutton L, Gunnell D, Bennewith O, Kapur N. Co-proxamol and suicide: preventing the continuing toll of overdose deaths. Qjm-An International Journal of Medicine. 2005;98: 159–70. 10.1093/qjmed/hci026 15728397

[43] Corcoran P, Reulbach U, Keeley HS, Perry IJ, Hawton K, Arensman E. Use of analgesics in intentional drug overdose presentations to hospital before and after the withdrawal of distalgesic from the Irish market. BMC Clinical Pharmacology. 2010;10. 10.1186/1472-6904-10-6 PMC2858125 20298551

[44] Hawton K, Bergen H, Simkin S, Brock A, Griffiths C, Romeri E, et al Effect of withdrawal of co-proxamol on prescribing and deaths from drug poisoning in England and Wales: time series analysis. British Medical Journal. 2009;338. 10.1136/bmj.b2270 PMC3269903 19541707

[45] Sandilands EA, Bateman DN. Co-proxamol withdrawal has reduced suicide from drugs in Scotland. British Journal of Clinical Pharmacology. 2008;66: 290–3. 10.1111/j.1365-2125.2008.03206.x 18489609 PMC2492929

[46] Segest E, Harris CN, Bay H. Dextropropoxyphene deaths in Denmark from the health authority point of view. Med Law. 1993;12: 41–52. 8377607

[47] Gusmao R, Quintao S, McDaid D, Arensman E, Van Audenhove C, Coffey C, et al Antidepressant Utilization and Suicide in Europe: An Ecological Multi-National Study. Plos One. 2013;8. 10.1371/journal.pone.0066455 PMC3686718 23840475

[48] Isacsson G, Bergman U, Rich CL. Epidemiological data suggest antidepressants reduce suicide risk among depressives. Journal of affective disorders. 1996;41: 1–8. 8938199 10.1016/0165-0327(96)00050-x

[49] Grunebaum MF, Ellis SP, Li S, Oquendo MA, Mann JJ. Antidepressants and suicide risk in the United States, 1985–1999. The Journal of clinical psychiatry. 2004;65: 1456–62. 15554756 10.4088/jcp.v65n1103

[50] Baca-Garcia E, Diaz-Sastre C, Saiz-Ruiz J, de Leon J. How safe are psychiatric medications after a voluntary overdose? Eur Psychiatry. 2002;17: 466–70. 12504263 10.1016/s0924-9338(02)00706-x

[51] Bhaskaran J, Johnson E, Bolton JM, Randall JR, Mota N, Katz C, et al Population trends in substances used in deliberate self-poisoning leading to intensive care unit admissions from 2000 to 2010. The Journal of clinical psychiatry. 2015;76: e1583–9. 10.4088/JCP.14m09568 26717534

[52] Paulozzi LJ, Kilbourne EM, Desai HA. Prescription drug monitoring programs and death rates from drug overdose. Pain medicine (Malden, Mass). 2011;12: 747–54. 10.1111/j.1526-4637.2011.01062.x 21332934

[53] Haffajee RL, Jena AB, Weiner SG. Mandatory Use of Prescription Drug Monitoring Programs. Jama-Journal of the American Medical Association. 2015;313: 891–2. 10.1001/jama.2014.18514 PMC4465450 25622279

[54] Hooper S, Bruno R, Sharpe M, Tahmindjis A. Alprazolam prescribing in Tasmania: a two-fold intervention designed to reduce inappropriate prescribing and concomitant opiate prescription. Australasian psychiatry: bulletin of Royal Australian and New Zealand College of Psychiatrists. 2009;17: 300–5. 19585293 10.1080/10398560902998626

[55] Buckley NA, Whyte IM, Dawson AH, McManus PR, Ferguson NW. Correlations between prescriptions and drugs taken in self-poisoning. Implications for prescribers and drug regulation. The Medical journal of Australia. 1995;162: 194–7. 7877541

